# Evaluation and Differential Diagnosis of a Genetic Marked Brucella Vaccine A19ΔvirB12 for Cattle

**DOI:** 10.3389/fimmu.2021.679560

**Published:** 2021-06-07

**Authors:** Jianghua Yang, Chuanyu He, Huan Zhang, Mengzhi Liu, Hailong Zhao, Lisong Ren, Dongling Wu, Fangyuan Du, Baoshan Liu, Xiaohu Han, Sun He, Zeliang Chen

**Affiliations:** ^1^ Key Laboratory of Livestock Infectious Diseases in Northeast China, Ministry of Education, College of Animal Science and Veterinary Medicine, Shenyang Agricultural University, Shenyang, China; ^2^ Tecon Biological Co. Ltd., Urumqi, China; ^3^ Beijing Advanced Innovation Center for Soft Matter Science and Engineering, Beijing University of Chemical Technology, Beijing, China; ^4^ Brucellosis Prevention and Treatment Engineering Technology Research Center of Inner Mongolia Autonomous Region, Inner Mongolia University for Nationalities, Tongliao, China; ^5^ School of Public Health, Sun Yat-sen University, Guangzhou, China

**Keywords:** protein chip technology, *B. abortus*, A19ΔvirB12, proteomics, differential diagnosis, attenuation mechanism

## Abstract

*Brucella abortus* is an important zoonotic pathogen that causes severe economic loss to husbandry and poses a threat to human health. The *B. abortus* A19 live vaccine has been extensively used to prevent bovine brucellosis in China. However, it is difficult to distinguish the serological response induced by A19 from that induced by natural infection. In this study, a novel genetically marked vaccine, A19ΔvirB12, was generated and evaluated. The results indicated that A19ΔvirB12 was able to provide effective protection against *B. abortus* 2308 (S2308) challenge in mice. Furthermore, the safety and protective efficacy of A19ΔvirB12 have been confirmed in natural host cattle. Additionally, the VirB12 protein allowed for serological differentiation between the S2308 challenge/natural infection and A19ΔvirB12 vaccination. However, previous studies have found that the accuracy of the serological detection based on VirB12 needs to be improved. Therefore, we attempted to identify potential supplementary antigens with differential diagnostic functions by combining label-free quantitative proteomics and protein chip technology. Twenty-six proteins identified only in S2308 were screened; among them, five proteins were considered as potential supplementary antigens. Thus, the accuracy of the differential diagnosis between A19ΔvirB12 immunization and field infection may be improved through multi-antigen detection. In addition, we explored the possible attenuation factors of *Brucella* vaccine strain. Nine virulence factors were downregulated in A19ΔvirB12. The downregulation pathways of A19ΔvirB12 were significantly enriched in quorum sensing, ATP-binding cassette transporter, and metabolism. Several proteins related to cell division were significantly downregulated, while some proteins involved in transcription were upregulated in S2308. In conclusion, our results contribute to the control and eradication of brucellosis and provide insights into the mechanisms underlying the attenuation of A19ΔvirB12.

## Introduction

Brucellosis is an important reemerging zoonosis that causes tremendous economic losses in animal husbandry and presents a significant risk to public health. The pathogen of brucellosis is *Brucella*, a small gram-negative coccobacillus lacking spores. Several *Brucella* species have been found to be pathogenic to humans, including *B. melitensis*, *B. abortus*, *B. suis*, *and B. canis*. Transmission can occur through direct contact with body fluids of infected animals, ingestion of contaminated dairy products, and aerosol (1–3). In the absence of an effective vaccine protecting humans from *Brucella* infection, the research and development of animal vaccines will be beneficial to both animals and humans (4, 5).

The *B. abortus* 2308 strain A19 vaccine has been widely employed in China and offers a high level of protection for cattle. However, the antibody response induced by the O-side chain of the A19 vaccine interferes with serological diagnosis, which hardly distinguishes between vaccinated and infected animals (6). Undoubtedly, this shortcoming of traditional vaccines impairs efforts to control and eradicate infectious diseases. Fortunately, the development and application of gene-deleted vaccines can overcome this limitation (7, 8). It is well known that the use of gene-deleted vaccines, such as gE-null marker strains, has led to great progress in the eradication of Pseudorabies virus in some areas (9).

Therefore, we generated the genetically marked vaccine A19ΔvirB12 by knocking out *virB12* gene in this study. The VirB12 protein, a component of the type IV secretion system (T4SS), has been shown to be a potential marker for the serological diagnosis of brucellosis (10). The results indicated that A19ΔvirB12 has similar virulence to that of its parent strain in mice. Immunization of mice with A19ΔvirB12 conferred protection against challenge with the virulent *B. abortus* 2308 (S2308). Furthermore, the safety and protective efficacy of A19ΔvirB12 were confirmed in cattle. VirB12-based iELISA can differentiate the vaccination from S2308 challenge/natural infections. However, previous studies have shown that the sensitivity and specificity of serological detection based on VirB12 needs to be improved (10, 11). In this regard, new antigens should be identified and combined with VirB12 to improve the accuracy of differential diagnosis. Proteomics has become an indispensable tool for comparing the differences of protein expression profiles between virulent and attenuated strains (12–14). Moreover, protein microarrays are an effective method for discovering antigen-specific antibody responses and identifying novel antigens. The two methods were combined in this study to screen for supplementary differential diagnosis antigens. In addition, based on the difference of proteomics between A19ΔvirB12 and S2308, we explored factors that may lead to significant differences in virulence between wild-type and vaccine strains.

## Materials and Methods

### Ethics Statement

BALB/c female mice (age, 7 weeks) were acquired from Tecon Biological Co., Ltd. (License number: SCXK2003-0002, Urumqi, China) and were housed in microisolator cages containing wood shavings in laminar flow racks. Mice inoculated with live attenuated *Brucella* vaccine strains were maintained under biosafety level-2 (BSL-2) containment and those inoculated with S2308 were maintained under BSL-3 containment. All mice were acclimated to the facility for 5 days prior to vaccination or infection, and were maintained on a 12-h/12-h light-dark cycle with free access to food and filtered water. Female Xinjiang Brown cattle (age, 3-8 months) with no prior infection with *Brucella* were selected for analysis in this study and housed in an outdoor and restricted access isolation facility. One to two weeks prior to the challenge, the cattle were transferred to a BSL-3 containment facility, where they were housed throughout the study duration. All animal experiments were strictly performed in accordance with the Experimental Animal Regulation Ordinances (2017) formulated by the China National Science and Technology Commission. The protocol was approved by the Committee on Ethics and Welfare of Experimental Animals of Tecon biological Co., Ltd.

### Cells, Plasmids and Bacterial Strains

Es*cherichia coli* (*E. coil*) DH5α and BL21 (DE3) cells were purchased from Transgen (Beijing, China). The plasmid pUC19-SacB was obtained from the Changchun Veterinary Research Institute (Changchun, China). The plasmid pET-28a was purchased from Transgen (Beijing, China). The virulent strains of S2308 and *B. melitensis* M28, and A19 vaccine strain were obtained from Tecon Biological Co., Ltd. (Urumqi, China). The A19ΔvirB12 mutant was constructed as previously described, with some modifications (15). Briefly, the upstream and downstream sequences of *virb12* were amplified from S2308 genomic DNA. Subsequently, the two products were ligated to one another *via* overlapping PCR, and the ligated product was inserted into pUC19-SacB to generate the pUC19-SacB-ΔvirB12 plasmid. Competent A19 was subjected to electroporation with pUC19-SacB-ΔvirB12, and then A19ΔvirB12 was isolated, identified, and used in this study.

### Evaluation of Virulence of A19ΔvirB12 Mutant in BALB/c Mice

Groups of female BALB/c mice (age, 7 weeks; n=4 per group) were injected intraperitoneally (i.p.) with 5.0×10^5^ colony formation units (CFU) of A19 or A19ΔvirB12 in 0.2 ml of phosphate-buffered saline (PBS; pH 7.2). The PBS-inoculated group served as a control. At 2, 4, 6, 8, and 10 weeks post-infection (wpi), mice were sacrificed, the spleens were removed, weighed, and then homogenized. Tissue homogenates were serially diluted with PBS, plated on tryptic soy agar (Becton, Dickinson and Company, USA) and incubated for 3 to 5 days at 37°C. The number of CFUs per spleen was measured.

### Protective Efficacy Evaluation and Cytokine Response Detection in BALB/C Mice

To evaluate the protective efficacy of A19ΔvirB12, groups of four female BALB/c mice were inoculated i.p. with 5.0 x 10^5^ CFU A19ΔvirB12 or 5.0 x 10^5^ CFU A19. Mice inoculated with PBS served as control. At 9 weeks post-vaccination (wpv), the mice were challenged i.p. with 5.0 x 10^5^ CFU (200 µl) of virulent S2308 per mouse. Three weeks after virulent challenge, all mice were euthanized by CO_2_ asphyxiation, and the spleens were collected and weighed. In addition, the levels of gamma interferon (IFN-γ) and interleukin-4 (IL-4) in mice serum were determined at 2, 4, 6, 8, 10 wpv and 3 weeks post-challenge(wpc) using the mouse IL-4 ELISA kit and the IFN-γ ELISA kit, according to the manufacturer’s instructions (Cloud-Clone, Corp., USA). Briefly, the ELISA plate had been pre-coated with antibodies specific to IL-4/IFN-γ. Standards or samples were then added to the wells with a biotin-conjugated antibody specific to IL-4/IFN-γ. Then, Avidin conjugated to Horseradish Peroxidase was added to each well and incubated. Subsequently, TMB substrate solution was added and the reaction was terminated by the addition of sulphuric acid solution. The concentration of IL-4/IFN-γ was then measured according to the standard curve.

### Evaluation of A19ΔvirB12 in Cattle

Groups of 12 female Xinjiang Brown cattle (age, 3-8 months) with no prior infection with *Brucella* were vaccinated subcutaneously with 6.0×10^10^ CFU of A19ΔvirB12 or 6.0×10^10^ A19. Control unimmunized cattle were injected with equivalent PBS. The rectal temperature of all the cattle was measured at the same time before and after immunization. At 5 weeks post-immunization, groups of five cattle were intraconjuctivally challenged with *B. melitensis* M28 at 10^9^ CFU (50 μl of inoculum per eye). Sixty days later, the cattle were euthanized and their submandibular lymph nodes, anterior shoulder lymph nodes, inguinal lymph nodes, and spleen were removed (0.3-0.5g) and washed with sterile PBS 3-5 times. The weighed tissues were placed into a sterile tube with two steel balls and 1 ml PBS, and then homogenized. The tissue homogenate (100 μl) was spread on *Brucella* selective medium dish and incubated in a 37°C incubator under 5% CO_2_ for 3-5 days. The *Brucella* genus-specific gene *bcsp31*-based PCR method (16) was used to detect the suspected *Brucella* colonies on the petri dish. The positive colonies amplified *via* bcsp31-PCR were further analyzed through classical AMOS-PCR, which was previously developed to identify and differentiate specific *Brucella* species. The typical *B. melitensis* yielded a specific band of 731 bp, and *B. abortus* yielded a specific band of 498 bp (17). To evaluate the safety of A19ΔvirB12 in pregnant cattle, A19ΔvirB12 immunization was carried out at the end of May in three local experimental pastures, and the calving situation of pregnant cattle was recorded in detail from March to November in the same year. The pregnancy status of the cattle was determined through rectal palpation.

### VirB12-Based iELISA in BALB/c Mice and Cattle

The recombinant protein VirB12 was produced as previously described with some modifications (15). Open reading frames (ORFs) of VirB12 were amplified through PCR using S2308 as a template (14). Subsequently, the amplified DNA fragments were cloned into the pET-28a vector and expressed in *E. coli* BL21 (DE3) cells (Transgen, Beijing). The recombinant protein VirB12 was confirmed through SDS-PAGE and western blotting. Then, the recombinant protein VirB12 was purified using affinity chromatography. The concentration of purified proteins was determined using a BCA kit (Thermo Fisher Scientific, Waltham, MA, USA) according to the manufacturer’s instructions. After determining the concentration, VirB12 served as coating antigen for indirect enzyme-linked immunosorbent assay (iELISA). Serum samples used for antibody detection were obtained from mice inoculated with S2308 or A19ΔvirB12, and cattle with natural infection or A19ΔvirB12 vaccination. The iELISA procedure was carried out as previously described (18).

### Collection of Sera and Fabrication of Protein Chip

Nine female Xinjiang Brown cattle (age, 3-8 months) were randomly assigned to either the immunized group (n=4) or the challenged group (n=5). The immunized group was subcutaneously injected with 6.0×10^10^ CFU of A19ΔvirB12, while the challenged group was intraconjuctivally inoculated with 1.0×10^9^ of S2308 (50 μl of inoculum per eye). Immunized sera were collected at 7, 14, 21, 28, 35, 42, 49, 56, and 63 days post-vaccination, and challenged sera were collected at 7, 14, 21, and 45 days post-infection.

The fabrication of the protein chip was performed as previously described with some modifications. Briefly, the ORFs of selected proteins were obtained using GenBank sequences NC_007618.1"> and NC_007624.1"> and amplified using gene-specific primers containing a 33 bp “adapter” sequence, which is homologous to the cloning sites of the linearized pXT7 vector. The genomic DNA of S2308 served as a template, and PCR products were visualized *via* agarose gel electrophoresis. A mixture containing linearized pXT7 vector and PCR-generated ORF fragment was transformed into *E. coli* DH5α cells and incubated overnight at 37°C. Subsequently, the recombinant plasmids were extracted using a QIAprep 96 Turbo Kit (Qiagen). The recombinant plasmids were expressed in *E. coli* using an *in vitro* transcription-translation (IVTT) reaction system (RTS Kit, Roche) according to the manufacturer’s instructions. The reaction of the negative control was performed in the absence of DNA. The reaction solution was printed onto nitrocellulose coated glass FAST slides. Slides were blocked for 30 min and then incubated with diluted serum samples at 4°C overnight. The slides were then washed and incubated with labeled secondary antibodies. The slides were scanned using a confocal laser, and the signal intensity of individual spots was quantified using QuantArray software.

### Protein Sample Preparation for Mass Spectrometry (MS) Analysis

Whole cell protein samples of virulent S2308 and A19ΔvirB12 strain were used for proteome analysis. All operations related to live *Brucella* were carried out in BSL-3 labs, and each sample had 3 biological replicates. Protein samples were prepared as described previously with some modifications (19). Briefly, bacteria were cultured to logarithmic and stationary phases. The logarithmic and stationary phase of the virulent S2308 were named Logw and Staw, respectively, while the logarithmic and stationary-phase A19ΔvirB12 vaccine strain were named Logv and Stav, respectively. The pH values of all culture media were adjusted to 7.0. Cells were harvested and centrifuged at 7,000×g for 15 min at 4°C and washed three times with sterile PBS buffer. The bacteria were resuspended in lysis buffer and lysed by ultrasonication. The remaining debris were removed by centrifugation at 1,2000×g for 30 min at 4°C, and then the supernatant was collected and stored at −80°C before trypsin digestion.

The BCA kit (Thermo Fisher Scientific, Waltham, USA) was used to measure protein concentration. Dithiothreitol (DDT) was added as a reductant to a final concentration of 5 mM and the sample was incubated at 56°C for 30 min. After reduction, the solution was alkylated with 11 mM iodoacetamide for 15 min at room temperature in the dark. Trypsin was added to the alkylated protein solution at a ratio of 1:50 (trypsin: protein, w/w) at 37°C overnight. Finally, trypsin was added at a ratio of 1:100 (trypsin: protein, w/w) for another 4 h.

### HPLC Separation and LC–MS/MS Analysis

Peptides produced by trypsin digestion were separated by high pH reversed-phase HPLC using an Agilent 300 Extend C18 column (5 μm particle size, 4.6 mm ID, 250 mm length). Peptides were fractionated with a gradient of 2% to 60% acetonitrile under alkaline conditions (pH =9.0) into 60 fractions. These fractions were then merged into four fractions and dried under vacuum.

Peptides were dissolved using buffer A (containing 0.1% formic acid and 2% acetonitrile) and then separated *via* EASY-nLC 1200 ultra HPLC system. The gradient comprised an increase from 8% to 16% of buffer B (0.1% formic acid in 98% acetonitrile) for approximately 30 min, 18% to 32% for 25 min, and to 80% in 2 min, and hold at 80% for the last 3 min. The flow rate was maintained at 400 nl/min. After separation, peptides were subjected to NSI source and then analyzed using tandem MS/MS in Q ExactiveTM Plus (Thermo Fisher Scientific, Waltham, USA). The electrospray voltage applied was set to 2.0 kV. The m/z scan range of the full scan was set from 350 to 1,800, and intact peptides were detected in the Orbitrap with a resolution of 70,000. Peptides were selected for MS/MS using the NCE setting 28 and the fragments were detected in the Orbitrap with a resolution of 17,500. A data-dependent procedure that alternated between one MS scan followed by 20 MS/MS scans with 15.0s dynamic exclusion. Automatic gain control was set at 5E4. The fixed first mass was set as 100 m/z.

### Database Searching

A Maxquant search engine (v.1.5.2.8) was employed for processing the resulting MS/MS data. Tandem mass spectra were searched against the UniProt *B. abortus* (3,023 sequences) concatenated with a reverse decoy database. Trypsin/P was designated as the cleavage enzyme, allowing up to 2 missing cleavages. The mass tolerance for precursor ions was set as 20 ppm in the first search and 5 ppm in the main search, and the mass tolerance for fragment ions was set as 0.02 Da. Alkylation of cysteine was set as a fixed modification and variable modifications were oxidation of methionine, acetylation of the N-terminal of protein, and deamidation of asparagine. The FDR was adjusted to <1%.

### Bioinformatics Analyses

Functional annotation of proteins was carried out using various bioinformatics procedures, including Gene Ontology (GO), Kyoto Encyclopedia of Genes and Genomes (KEGG) and The Clusters of Orthologous Groups (COG). GO annotation proteome was derived from the UniProt-GOA database (http://www.ebi.ac.uk/GOA/). Firstly, the identified protein ID was converted to UniProt ID and then mapped to GO IDs by protein ID. If the identified proteins were not annotated by UniProt-GOA database, the InterProScan soft would be used to annotated protein’s GO functional based on protein sequence alignment method. KEGG connects known information on molecular interaction networks (20). Firstly, the protein’s KEGG database description was annotated using KEGG online service tools KAAS. Then the annotation result was mapped to the KEGG pathway database using KEGG online service tools KEGG mapper. COG (http://www.ncbi.nlm.nih.gov/COG/) system software program was used to determine the functional distribution of proteins (21).

### Statistical Analysis

Statistical analysis was performed using GraphPad Prism 7.0 (GraphPad Software, San Diego, CA, USA) and SPSS software (version 22.0, IBM, Armonk, NY, USA). Statistical analysis was performed by a two-tailed Student’s t test, Fisher’s exact test or Mann-Whitney U test; a *P*-value less than 0.05 was considered statistically significant.

## Results

### Evaluation of Virulence and Immune Responses of A19ΔvirB12 in Mice

**Figure 1:**
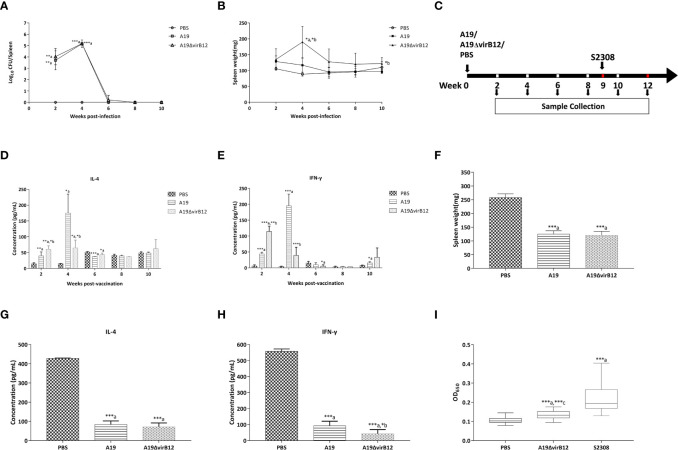
Characteristics of A19ΔvirB12 vaccination. To determine the virulence of A19ΔvirB12, female BALB/c mice were i.p. injected with 5 × 10^5^ CFU of A19ΔvirB12, A19, or PBS. Colonization levels **(A)** and spleen weights **(B)** were assessed at 2, 4, 6, 8, and 10 weeks post-infection. To determine the protective efficacy of immunization with A19ΔvirB12, female BALB/c mice were immunized i.p. with 5 × 10^5^ CFU of A19ΔvirB12 or A19 while the control group received 0.2 ml of PBS **(C)**. At 9 weeks post-vaccination, mice (n=5 per group) were challenged with 5 × 10^5^ CFU of virulent S2308 **(C)**. The levels of IL-4 **(D)** and IFN-γ **(E)** were measured at 2, 4, 6, 8, and 10 weeks post-vaccination. At 3 weeks post-challenge, the spleen weight **(F)**, and the levels of IL-4 **(G)** and IFN-γ **(H)** were measured. Immune response of VirB12 to A19ΔvirB12-immunized sera and challenged sera was shown in the **(I)**. Values was expressed as the means ± standard deviation (SD). Differences were determined by the two-tailed Student's t test or Mann-Whitney U test. The letter a in the **(A–I)** indicates statistically significant differences between A19 or A19ΔvirB12 and the PBS group; The letter b in the **(A–H)** indicates statistically significant differences between A19 and A19ΔvirB12; The letter c in the **(I)** indicates statistically significant differences between A19ΔvirB12 and S2308 (*P < 0.05; **P < 0.01; ***P < 0.001).

To evaluate the virulence of A19ΔvirB12 *in vivo*, BALB/c mice were infected with A19ΔvirB12 or its parental strain A19. As shown in **Figure 1A**, there was no significant difference in colonization levels in the spleen between A19ΔvirB12 and A19 (*P*>0.05) at 2, 4, 6, 8, or 10 wpi. The change of spleen weight in infected mice is closely related to the virulence of *Brucella* and the level of inflammatory response (22). Mice injected with A19ΔvirB12 showed significantly increased spleen weights compared with mice inoculated with A19 at 4 and 10 wpi (**Figure 1B**). Nevertheless, spleen weight tended to be normal in mice injected with A19ΔvirB12 or A19.

In order to determine the protection efficiency of A19ΔvirB12, the mice were vaccinated i.p. with A19ΔvirB12 or A19, and the PBS-inoculated group served as the control (**Figure 1C**). At 9 wpv, the mice were challenged i.p. with S2308 (**Figure 1C**). Compared with the PBS-inoculated group, the levels of IL-4 in A19 and A19ΔvirB12 vaccinated groups were significantly increased at 2,4 and 6 wpv (*P*<0.05; **Figure 1D**). As for IFN-γ, we obtained results similar to those of IL-4 at 2 and 4 wpv (**Figure 1E**). These results indicated that vaccination with the strain A19ΔvirB12 can induce obvious cellular immune response in mice. As shown in **Figure 1F**, mice immunized with A19ΔvirB12 or A19 exhibited lighter spleen weights compared with the PBS-inoculated group at 3 wpc (*P*<0.05). In addition, the concentrations of IL-4 and IFN-γ in mice vaccinated with A19ΔvirB12 or A19 were significantly lower than those of the PBS-inoculated control group (*P*<0.5; **Figures 1G, H**). These results indicated that compared to the PBS-inoculated group, the immunized mice may eliminate the wild-type strains more quickly. In addition, we compared the cytokine levels and spleen weights of mice at 3 wpc with those at 10 wpv (**Table S1**). The levels of IL-4 and IFN-γ in the A19-inoculation group at 3 wpc were higher than those at 10 wpv (*P<0.05*). In contrast, the cytokine levels and spleen weights of the A19ΔvirB12-immunized group at 3 wpc were similar to those at 10 wpv. Therefore, mice immunized with A19ΔvirB12 could recover to the pre-challenge level more quickly after challenge, suggesting that A19ΔvirB12 may have a better protective effect than A19 in mice.

### Evaluation of Differential Diagnosis Using VirB12 as a Test Antigen in Mice

To determine whether the VirB12 protein can be used as a diagnostic marker antigen, iELISA was employed to detect antibodies in A19ΔvirB12- and S2308-inoculated mice sera using the recombinant purified protein VirB12 as the coating antigen. As shown in **Figure 1I**, the levels of antibodies against VirB12 in the sera of *B. abortus* 2308-inoculated mice were significantly higher than those of A19ΔvirB12-inoculated mice (*P*<0.05). Therefore, VirB12 may be used to differentiate vaccination from a natural infection.

### Evaluation of A19ΔvirB12 in Cattle and Field Application

**Figure 2:**
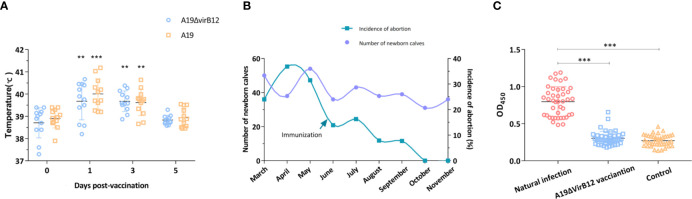
Evaluation of A19ΔvirB12 vaccination in cattle. **(A)** Changes of body temperature after immunization. **(B)** Incidence of cattle abortion by month before and after A19ΔvirB12 immunization. **(C)** Immune response of VirB12 to sera with A19ΔvirB12 immunization or natural infection. The arrow in **(B)** represents the time point of A19ΔvirB12 immunization. * in the **(A)** indicates statistically significant differences between the groups of A19ΔvirB12/A19 vaccination and pre-vaccination. Differences were determined by the two-tailed Student’s t test or Mann-Whitney U test (**P < 0.01; ***P < 0.001).

The results of mice experiments indicated that A19ΔvirB12 is a potential live attenuated vaccine with a differential diagnostic function. Subsequently, the safety and protective efficacy of A19ΔvirB12 in cattle were evaluated. After vaccination, the temperature of the cattle fluctuated within the normal temperature range (38-40°C; **Figure 2A**). At 1 and 3 days post-immunization, there was a slight increase in body temperature of the immunized groups. In general, the body temperature of cattle increased after vaccination, but soon returned to the pre-immunization level. The most dominant *Brucella* species in China was *B*. *melitensis*, which is the important pathogen responsible for sheep and cow abortion (23, 24). Thus, A19-immunized and A19ΔvirB12-immunized cattle were challenged with virulent *B*. *melitensis* M28 at 35 days post-immunization to evaluate the protective efficacy. At 60 days post-challenge, the clearance of M28 within the submandibular lymph nodes, anterior shoulder lymph nodes, inguinal lymph nodes, and spleen was evaluated. Compared with the control group, M28 was effectively eliminated in the A19ΔvirB12-immunized group (**Table 1**), suggesting that A19ΔvirB12 immunization can protect cattle against wild-type infection. In addition, the safety of A19ΔvirB12 was evaluated in large-scale pregnant cattle. Previous epidemiological studies have shown that the occurrence of cattle abortion is caused by multiple factors (25). In the same pasture, the occurrence of cattle abortion is mainly related to seasonal variation. Winter is the season with the lowest incidence of cattle abortion, and the incidence begins to increase in March and reaches a peak in summer (26, 27). In this study, we also observed an extremely high incidence of cattle abortion in spring. Fortunately, A19ΔvirB12 vaccination did not cause deterioration (**Figure 2B**), which may have played a role in curbing the increase in the incidence of cattle abortion, indicating that A19ΔvirB12 is safe in pregnant cattle. Similarly, the sera of naturally infected cattle and A19ΔvirB12-immunized cattle were analyzed through ELISA using VirB12 as a coating antigen. The results showed that the level of anti-VirB12 antibody in naturally infected cattle serum was significantly higher than that in A19ΔvirB12-immunized serum (**Figure 2C**). Therefore, the differential diagnostic function of the A19ΔvirB12 vaccine is supported by data from both mice and cattle.

**Table 1:** The effect of vaccine immunization on the elimination of virulent *Brucella*.

Detection	PBS (n/N)	A19 (n/N)	A19ΔvirB12 (n/N)
Bacterial culture^+^	5/5	4/5	4/5
*bcsp31*-PCR^+^	5/5	3/5	2/5
AMOS-PCR^+^	5/5	2/5	1/5

+, positive test result; n, number of cattle with the positive result; N, total number of cattle per group.

### Overview of Proteomics and Screening of Differentially Expressed Proteins Involved in Virulence

In order to discover specific proteins of S2308, a label-free proteomics approach was used to compared the protein expression profiles of S2308 and A19ΔvirB12. The screening process of target antigens is shown in **Figure S1**. First, whole proteins were extracted from A19ΔvirB12 and S2308 in the logarithmic and stationary phases, digested with trypsin, followed by LC–MS/MS analysis. In total, 2185 proteins were identified, of which 1999 were quantifiable (**Table S2**). The proteins were divided into two groups for analysis: Log w/v (Logw vs Logv) and Sta w/v (Staw vs Stav). With ratios of 1.50 and 0.67 as the cutoffs for differential up and downregulated expression, the expression levels of 154 proteins were found to increase in the logarithmic-phase S2308, while 121 proteins showed a decrease. In the stationary period, 66 upregulated proteins and 59 downregulated proteins were observed in S2308 (**Table S3**). The analysis of COG and GO for the DEPs is shown in **Figure S2**, **Tables S4** and **S5**.

*Brucella* has high pathogenicity to human and animals; however, spontaneous attenuated strains have low virulence and could provide immune protection for vaccinated animals against natural infection (28). In this study, we compared the protein expression profiles of S2308 and A19ΔvirB12 in order to explore the potential molecular mechanisms causing the virulence attenuation. We screened the upregulated virulence factors identified in S2308 using the Victors database (http://www.phidias.us/victors), an online database for virulence factors of pathogens (29). We observed that 8 and 3 virulence-related proteins were downregulated in the logarithmic and stationary phase of A19ΔvirB12, respectively (**Table 2**). Subcellular localization showed that most of these virulence proteins were located in the cytoplasm, followed by the cytoplasmic membrane. The results of COG functional analysis showed that these virulence proteins were mainly related to amino acid metabolism, transport, and transcription, followed by transcription. Both VjbR (encoded by *vjbR*) and urease subunit alpha 2(*ureC2*) were downregulated during the growth of A19ΔvirB12.

**Table 2:** Downregulated virulence-regulated proteins in A19ΔvirB12.

Gene name	Protein accession	Protein description	Ratio	Subcellular localization	COG category
**Downregulated virulence-related factor in logarithmic-phase A19ΔvirB12**					
**vjbR**	Q2YJ50	HTH-type quorum sensing-dependent transcriptional regulator VjbR	0.163	Unknown	K
**cydB**	Q2YKD5	Beta and gamma crystallin:Cytochrome bd ubiquinol oxidase, subunit II	0.158	CytoplasmicMembrane	C
**bacA**	Q2YMA1	Bacteroid development protein BacA	0.229	CytoplasmicMembrane	I
**pgm**	Q2YPS4	Phosphoglucomutase/phosphomannomutase:Phosphogluc omutase/phosphomannomutase C terminal:Phosphoglucomutase/phosphomannomutase	0.246	Cytoplasmic	G
**leuA**	Q2YRT1	2-isopropylmalate synthase	0.444	Cytoplasmic	E
**recA**	Q2YRU7	Protein RecA	0.486	Cytoplasmic	L
**ureG2**	Q2YQD6	Urease accessory protein UreG 2	0.193	Cytoplasmic	KO
**ureC2**	Q2YQD8	Urease subunit alpha 2	0.044	Cytoplasmic	E
**Downregulated virulence-related factor in stationary-phase A19ΔvirB12**					
**vjbR**	Q2YJ50	HTH-type quorum sensing-dependent transcriptional regulator VjbR	0.227	Unknown	K
**ureC2**	Q2YQD8	Urease subunit alpha 2	0.117	Cytoplasmic	E
**ureA2**	Q2YQE0	Urease subunit gamma 2	0.172	Cytoplasmic	E

E, represents amino acid transport and metabolism; C, represents energy production and conversion; I, represents lipid transport and metabolism; G, represents carbohydrate transport and metabolism; K, represents transcription; L, represents replication, recombination, and repair; O, represents posttranslational modification, protein turnover, chaperons.

### KEGG Enrichment of DEPs

The KEGG enrichment results of DEPs in the log w/v and Sta w/v groups are shown in **Table 3**. The upregulated pathways in the log w/v group were mainly enriched for ATP-binding cassette (ABC) transporters, quorum sensing (QS), and metabolism of multiple substances. The important role of ABC transporters and QS system in microbial virulence has been revealed (30, 31). In addition, there is also an important link between metabolism and virulence in *Brucella* (32). As for stationary-phase DEPs, the upregulated DEPs were also found to be enriched in the metabolism of multiple substances, including branched chain amino acids (BCAA; valine, leucine, and isoleucine), tryptophan, atrazine, and propanoate. The downregulated DEPs were found to be enriched in amino acid biosynthesis. This suggests that there are differences in degradation and synthesis of substances between the two strains in the stationary phase.

**Table 3:** KEGG pathways enrichment for DEPs in the logarithmic and stationary phases (*P*-value <0.05).

KEGG pathway	Number of proteins	*P*-value
**Up-regulated pathways in Log w/v**		
**map02010 ABC transporters**	18	0.0250
**map02024 Quorum sensing**	12	0.0086
**map00640 Propanoate metabolism**	6	0.0375
**map00280 Valine, leucine and isoleucine degradation**	6	0.0320
**map00340 Histidine metabolism**	5	0.0214
**map00052 Galactose metabolism**	3	0.0388
**map00740 Riboflavin metabolism**	3	0.0184
**Down-regulated pathways in Log w/v**		
**map00540 Lipopolysaccharide biosynthesis**	3	0.0113
**map00261 Monobactam biosynthesis**	2	0.0369
**Up-regulated pathways in Sta w/v**		
**map01120 Microbial metabolism in diverse environments**	9	0.0110
**map00640 Propanoate metabolism**	3	0.0272
**map00280 Valine, leucine and isoleucine degradation**	3	0.0248
**map00380 Tryptophan metabolism**	2	0.0454
**map00290 Valine, leucine and isoleucine biosynthesis**	2	0.0298
**map00791 Atrazine degradation**	2	0.0074
**Down-regulated pathways in Sta w/v**		
**map01230 Biosynthesis of amino acids**	8	0.0065

P-value was determined by Fisher’s exact test.

### Potential Supplementary Antigens for Differential Diagnosis

**Figure 3:**
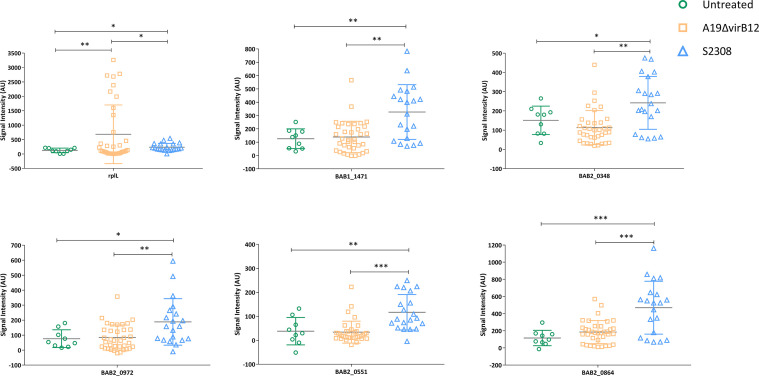
IgG response of 5 proteins obtained from sera of cattle inoculated with A19ΔvirB12 or S2308. Values were expressed as the means ± (SD). Differences were determined by two-tailed Student’s t test (*P < 0.05; **P < 0.01; ***P < 0.001).

**Figure 4:**
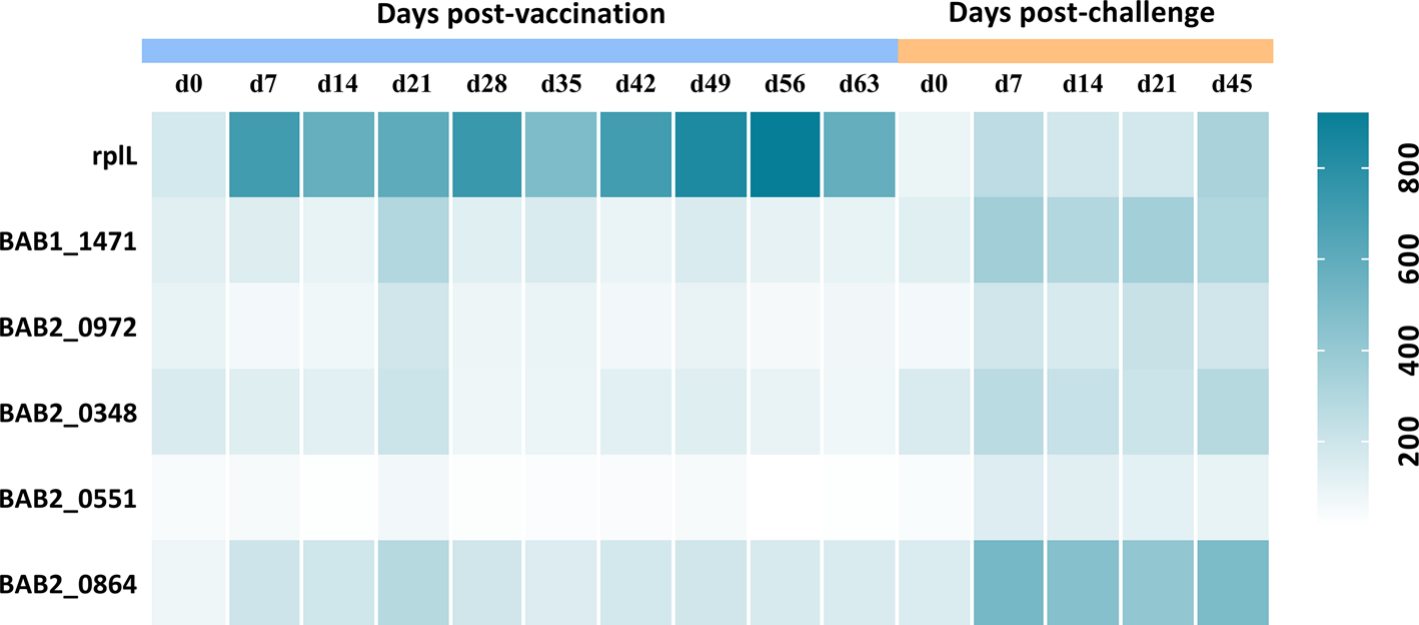
IgG response of 5 proteins obtained from sera of cattle inoculated with A19ΔvirB12 or S2308 at different time points. Values were expressed as average signal intensity of IgG response.

To find supplementary differential diagnostic antigens, we searched for proteins that were expressed only in S2308. A total of 26 proteins were identified and they are listed in **Table S6**. These proteins are located on the cytoplasmic membrane (10 proteins) or cytoplasm (9). Notably, three of them were T4SS proteins, including VirB4 (*virB4*), VirB9 (*virB9*), and VirB11 (*virB11*). We speculate that the absence of VirB12 may implicate the expression of other T4SS proteins. Subsequently, these specific proteins were constructed into protein arrays and interacted with cattle sera. The L7/L12(*rplL*) protein, known as *Brucella* antigen (33) was employed as a positive control, which showed no difference in the expression between the two strains in this study. Compared with immunized sera, a total of five proteins produced a stronger response intensity to challenged sera (*P*<0.05; **Figure 3**). The lower IgG responses of immunized sera suggested that these five proteins may have the potential for differential diagnosis between A19ΔvirB12 vaccination and natural infection. Subsequently, the IgG antibody kinetics of the cattle sera against these proteins were analyzed (**Figure 4**). The heat map showed directly that the challenged sera induced an obvious IgG response against these proteins, compared with the immunized sera. However, there seemed to be no regularity in the response of challenged sera to these proteins at different time points.

## Discussion

*B. abortus* infection causes rapid activation of innate immunity, including phagocytosis by professional phagocytes, such as macrophages, dendritic cells, and neutrophils. The activation of pattern recognition receptors by pathogen-associated pattern molecules and the secretion of cytokines are key factors in the elimination of *Brucella (* 34, 35). IFN-γ is required for resistance to brucellosis and is closely related to the elimination of *Brucella*. On the contrary, IL-4 antagonizes IFN-γ mediated antibacterial activity, and promotes chronic infection. This may be due to the interaction between host and *Brucella (* 36). *Brucella* infection has caused great economic losses, affecting many livestock, wildlife and human. Vaccine immunization is still a necessary strategy for the prevention and control of brucellosis. The licensed *Brucella* vaccines are live attenuated vaccines, which retain the invasion process similar to that of the wild-type strains, but are easier to be eliminated by the immune system and can induce the production of efficient humoral and cellular immune responses in immunized animals (37). However, these vaccines have obvious limitations in the control and eradication of brucellosis (28). At present, the development of gene-deleted vaccines allows for serological differential diagnosis between vaccination and field infections (38). Here, the *virB12* mutant was derived from *B. abortus* 2308 strain A19. Firstly, the deletion of *virB12* gene did not affect the replication ability of A19 within the mice. In addition, vaccination with A19ΔvirB12 could elicit obvious cellular immune response and provide good protection efficacy against a challenge with S2308 in mice. A19ΔvirB12 vaccinated mice induce production of IFN-γ and IL-4. However, low levels of IL-4 were induced in mice vaccinated with another commonly used vaccine, *B. abortus* RB51, indicating that different vaccine strains cause different protective mechanism. Furthermore, the safety and protection efficiency of A19ΔvirB12 were confirmed in natural host cattle. *Brucella* infection in cows and ewes is a public health issue and *B. melitensis* has become the dominant species in China. Although goats and sheep are the preferential host for *B. melitensis*, this pathogen has become a cause for *Brucella* outbreaks in cattle (24, 39, 40). Our results showed that vaccination with A19ΔvirB12 can confer effective protection in cattle against the challenge with *B. melitensis* M28 and is safe for pregnant cattle. What’s more, VirB12 allowed for the serological differentiation between S2308 challenge/natural infection and A19ΔvirB12 vaccination. Recently, A19ΔvirB12 was approved and became the first brucellosis genetically marked vaccine in China (41).

However, the sensitivity and specificity of differential diagnosis based on a single protein need to be improved. We believe that detection of multiple antigens may improve the accuracy of differential diagnosis. Therefore, we attempted to identify novel antigens that could induce differential responses to A19ΔvirB12 vaccination and natural infection. Using a proteomics approach, the protein expression profiles of S2308 and A19ΔvirB12 were compared, and 26 proteins were identified only in S2308. Currently, most proteomics studies focus on differential regulatory proteins. In this study, we focused on the proteins only expressed in the virulent strain, which may have the potential for differential diagnosis. Protein-chip technology was used to further confirm the immune response of these proteins; the IgG response level of challenged sera against five proteins was higher than that of immunized sera in cattle. This suggests that A19ΔvirB12 immunization and field infection may be more accurately distinguished by detecting multiple antigens. Differential vaccines combined with companion diagnostic methods facilitate the control and eradication of epidemic diseases (38). Future research should focus on the development of corresponding diagnostic approaches. In general, we innovatively combined the proteomics approach with the protein array technology in this study, which provides a reference strategy for screening target proteins for the differential diagnosis of other pathogens.

In addition, virulence attenuation mechanisms of vaccine strains are the focus of many studies (42–44). There was no significant difference at the genomic level between the S2308 and A19 vaccine strains (45, 46). Therefore, proteomics studies may contribute to the understanding of the mechanisms causing great differences in virulence between the virulent and attenuated strains. In this study, potential attenuation mechanisms of A19ΔvirB12 were analyzed as follows.

First, some downregulated virulence factors in A19ΔvirB12 were identified (**Table 2**). VjbR protein (*vjbR*), a member of the LuxR-type QS regulators, was induced in both the logarithmic and stationary phase of S2308. Previous studies have shown that VjbR is involved in regulating the expression of virulence factors during the early stage of intracellular infection (47, 48). Urease, another factor associated with the survival of *Brucella* was also affected. In the process of gastrointestinal tract infection, the presence of urease, a nickel-binding protein, enhances the survival capability of pathogenic microorganisms in acidic environments, including *Brucella* (49–51). *Brucella* contains two urease operons, ure1 and ure2, and the latter contains the genes of ure ABCEFGDT involved in the transport of urea and nickel (50, 52). In this study, ureC2 and ureG2, contained in the ure2 operon, were more abundant at the protein level in the logarithmic phase of S2308, while ureC2 and ureA2 were found to be induced in the stationary phase of S2308 (52). In addition, several virulence gene products were upregulated in the logarithmic phase of S2308, including *cydB*, *bacA*, *recA*, *pgm*, and *leuA*. *cydB* is responsible for encoding cytochrome bd oxidase. The absence of *cydB* increases the sensitivity of *Brucella* to environmental stresses and affects its intracellular survival (53). Similarly, the bacteroid development protein BacA(*bacA*) is required for *Brucella* to establish chronic infection in mice (54). Intracellular pathogens inevitably encounter a series of adverse conditions during their life cycle. Timely repair of DNA damage is particularly vital for the intracellular survival of *Brucella* (55). RecA protein (*recA*) is an important protein involved in DNA repair pathways. There is no doubt that the lack of RecA reduces the ability of *Brucella* to deal with DNA damage (56). *pgm* is responsible for the production of phosphoglucomutase, catalyzing the conversion of glucose-6-phosphate to glucose-1-phosphate. Mutation of *pgm* that is related to the loss of O-antigen generates a rough phenotype that is usually less virulent (57). Another metabolic enzyme considered to be a virulence factor of *Brucella* was 2-isopropylmalate synthase (*leuA*), which is essential for the survival of *Brucella* within macrophages (29).

The QS pathway was found to be enriched significantly in the logarithmic phase of S2308 compared with that of A19ΔvirB12 (**Table 3**). QS is a cell-cell communication system involved in monitoring bacterial populations. First, bacteria produce small extracellular signaling molecules that accumulate gradually as the number of bacteria increases. When the concentration of signaling molecules reaches the threshold of detection limit, bacteria respond and synchronously regulate the expression of many genes. QS systems are involved in multiple bacterial processes that are beneficial for survival, such as biofilm formation, virulence factor secretion, and bioluminescence production (58, 59). In view of the role of the QS system in the regulation of virulence factors, it has become a new therapeutic target for some bacterial diseases (60). The significant enrichment of the QS pathway in S2308 may be one of the main reasons for the significant differences in virulence and intracellular survival between the marked vaccine strain and the virulent strain.

In addition, some proteins identified as being downregulated were associated with ABC transporters in the logarithmic phase of A19ΔvirB12 (**Table 3**). ABC transporters, a large group of membrane protein complexes, are responsible for transmembrane transport of multiple nutrients, accompanied by the hydrolysis of ATP (61). Previous studies have indicated that ABC transporters are related to virulence in some bacteria by transporting various nutrients and metal ions as well as promoting adhesion (62–64). Additionally, a previous study has indicated that the absence of an ABC transporter leads to increased susceptibility of *B. melitensis* to polymyxin B (65). In this work, the upregulated ABC transporters were mainly involved in the uptake of amino acids, especially BCAA (valine, leucine, and isoleucine). Amino acids not only provide energy for protein synthesis, but are also precursors of many important metabolites, such as ammonia, ketone bodies, and glucose (66). Moreover, BCAA uptake is closely related to the pathogenicity of pathogenic bacteria (67). Some intracellular pathogens rely on BCAA transport proteins to support their growth within host cells (68–70). Thus, decreased BCAA utilization may lead to lower virulence in A19ΔvirB12.

This group of proteins was associated with cell division. Interestingly, some key proteins participating in cell division were observed to be more abundant in A19ΔvirB12 *in vitro* culture (**Figure S2** and **Table S4**). Cell division is a complex process that requires the participation and coordination of a large number of proteins. The cell division protein ZapA is involved in the formation of a septum called Z-ring in the middle of the cell (71). Min proteins (MinE, MinC, and MinD) play a role in the right placement of Z-ring for equal cell distribution (72). The cytoplasmic ATPase FtsE is involved in promoting septal ring constriction. The cell division coordinator CpoB is required for the coordination of peptidoglycan synthesis and outer membrane constriction (73). Tyrosine recombinase XerD facilitates the disaggregation of chromosome dimers (74). In the logarithmic phase, ZapA, XerD, and CpoB were downregulated in S2308 compared with A19ΔvirB12 at the protein level. In addition, decreased expression levels of MinE and FtsE were observed in the stationary phase of S2308. We assumed that A19ΔvirB12 may have a higher growth rate. Indeed, higher growth rate leads to more material consumption. Especially in the stationary growth period, accompanied by resource depletion and increased levels of toxic metabolites, limiting reproduction level is a wise choice. Notably, in the early stage of the intracellular life cycle, growth arrest was observed in S2308 within the endosomal *Brucella*-containing vacuole (75). Thus, it may be an important survival strategy for S2308 to limit the level of cell reproduction when necessary.

Based on the COG analysis, 13 and 7 upregulated proteins related to transcription were found in the logarithmic and stationary phase of S2308, respectively. Most of them were bacterial regulatory proteins belonging to different families, such as ArsR, AsnC, and MerR families. Within the group of transcriptional regulators, one protein with σ70 factor activity was induced in S2308 during the logarithmic phase. σ factors are required for initiation of transcription at promoters, and most σ factors belong to the σ70-family. The existence of σ factor is important for bacteria to sense and respond to environmental changes in time (76). Thus, active transcriptional regulation, timely response, and adaptation to environmental changes may be key factors in the pathogenicity of virulent *Brucella* strains.

## Conclusion

In the present study, A19ΔvirB12 was successfully generated. The safety and protective efficacy of A19ΔvirB12 have been confirmed in mice and cattle. VirB12 protein has potential as a diagnostic antigen for the differentiation of A19ΔvirB12-immunization from field infection. We screened for supplementary antigens by combining proteomics and protein chip technology to optimize the accuracy of the differential diagnosis of the VirB12-based method. Among the 26 proteins identified only in S2308, five proteins induced a higher IgG response to challenged sera than immune sera. In addition, we analyzed virulence-related proteins and pathways between S2308 and A19ΔvirB12. The upregulation of virulence factors and QS system, active intake of nutrients, control of reproduction, timely response to environmental change, and induction of virulence factors may mediate the high pathogenicity of S2308. In other words, the difference in virulence is caused by multiple factors. The exploration of *Brucella* virulence will provide some potential targets for the treatment of brucellosis.

## Data Availability Statement

The datasets presented in this study can be found in online repositories. The names of the repository/repositories and accession number(s) can be found below: https://www.ebi.ac.uk/pride/archive/, PXD022031.

## Ethics Statement

The animal study was reviewed and approved by Committee on Ethics and Welfare of Experimental Animals of Tecon biological Co., Ltd.

## Author Contributions

ZC, SH, and BL conceived and designed the experiments and ML, HZhao, LR, FD and DW performed the experiments. XH, HZhan and CH carried out revision of manuscript. JY analyzed the data and draft the manuscript. All authors contributed to the article and approved the submitted version.

## Funding

This work was supported by the State Key Program of National Natural Science of China (U1808202), NSFC International (regional) cooperation and exchange program (31961143024), National Key Research and Development Program Projects (2017YFD0500901, 2017YFD0500305, 2016YFC1200100), the National Key Program for Infectious Disease of China (2018ZX10101002-002), Key Program of Inner Mongolia (2019ZD006).

## Conflict of Interest

Authors CH, ML, HZ, LR, DW, and SH were employed by Tecon Biological Co., Ltd.

The remaining authors declare that the research was conducted in the absence of any commercial or financial relationships that could be construed as a potential conflict of interest.

## Supplementary Material

Supplementary Figure 1 A brief screening process of supplementary differential diagnosis antigens for A19ΔvirB12. First, whole proteins were extracted from A19ΔvirB12 and S2308 in the logarithmic and stationary phases, digested with trypsin, followed by LC–MS/MS analysis. The protein expression profiles of A19ΔvirB12 and S2308 were compared and analyzed. Subsequently, the proteins identified only in S2308 were screened. Finally, protein chip technology was used to find the proteins that have the potential to distinguish immunized and challenged sera of cattle.

Supplementary Figure 2 COG and GO analysis of DEPs. **(A)** COG analysis for DEPs in the group of Log w/v. **(B)** COG analysis for DEPs in the group of Sta w/v. **(C)** GO analysis for DEPs in the group of Log w/v. **(D)** GO analysis for DEPs in the group of Sta w/v.

Supplementary Table 1 Comparison of cytokine level and spleen weight of mice before and after challenge. Differences were determined by the two-tailed Student’s t test.

Supplementary Table 2 Identified results of mass spectrometry.

Supplementary Table 3 Differentially expressed proteins in the groups of Log w/v and Sta w/v.

Supplementary Table 4 COG analysis for DEPs in the groups of Log w/v and Sta w/v.

Supplementary Table 5 GO analysis for DEPs in the groups of Log w/v and Sta w/v.

Supplementary Table 6 Specific proteins identified only in S2308.
