# Supplementary figures and images for: Evaluation and Differential Diagnosis of a Genetic Marked Brucella Vaccine A19ΔvirB12 for Cattle
Source: Front Immunol. 2021 Jun 7;12:679560. doi: 10.3389/fimmu.2021.679560 (PMC8215367; doi:10.3389/fimmu.2021.679560)

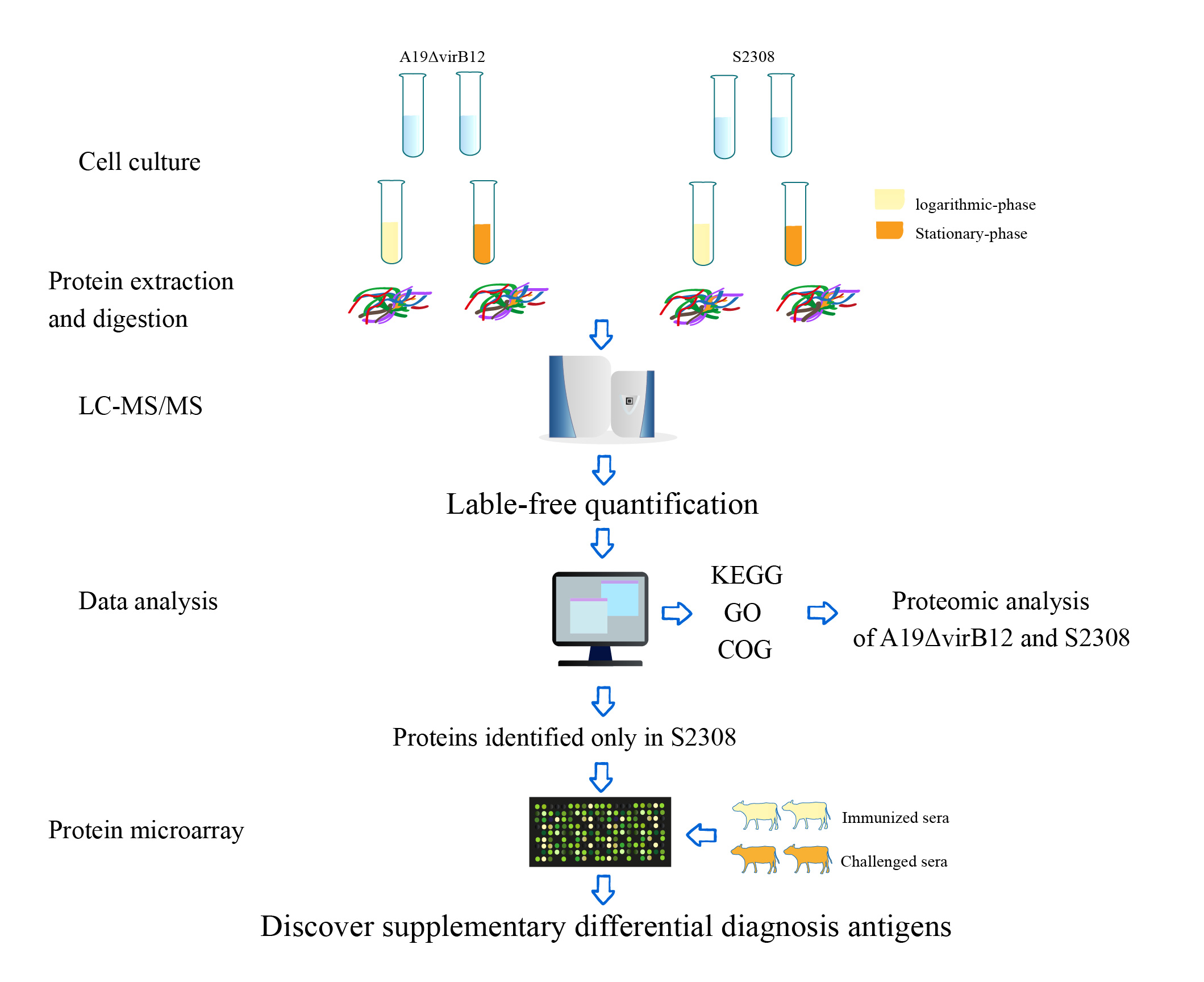

Supplement: Supplementary Figure 1 — A brief screening process of supplementary differential diagnosis antigens for A19ΔvirB12. First, whole proteins were extracted from A19ΔvirB12 and S2308 in the logarithmic and stationary phases, digested with trypsin, followed by LC–MS/MS analysis. The protein expression profiles of A19ΔvirB12 and S2308 were compared and analyzed. Subsequently, the proteins identified only in S2308 were screened. Finally, protein chip technology was used to find the proteins that have the potential to distinguish immunized and challenged sera of cattle. [file Image_1.jpeg]

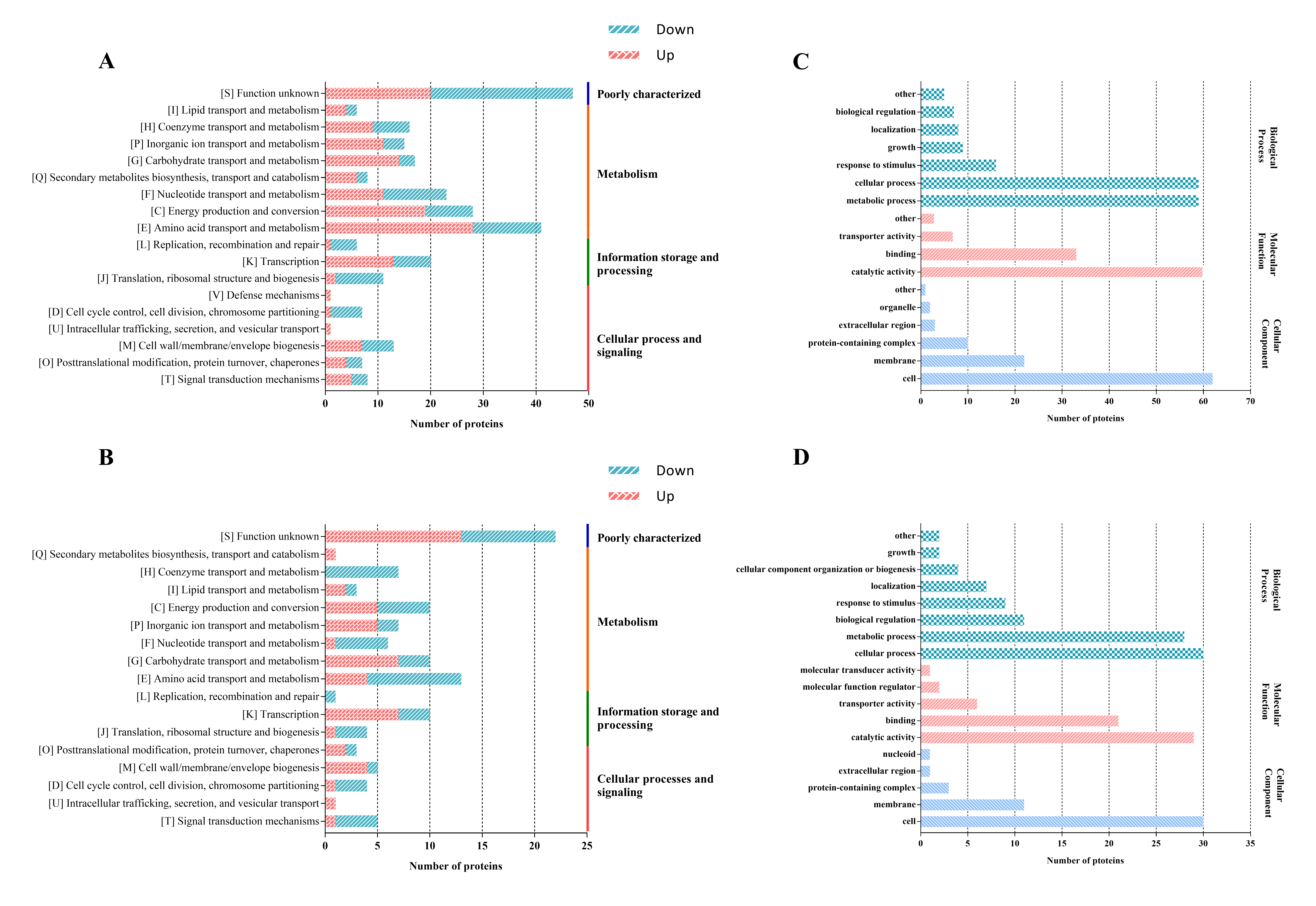

Supplement: Supplementary Figure 2 — COG and GO analysis of DEPs. (A) COG analysis for DEPs in the group of Log w/v. (B) COG analysis for DEPs in the group of Sta w/v. (C) GO analysis for DEPs in the group of Log w/v. (D) GO analysis for DEPs in the group of Sta w/v. [file Image_2.tif]
